# Cutaneous epitheliotropic T-cell lymphoma in a donkey – a case report

**DOI:** 10.1186/s12917-022-03365-7

**Published:** 2022-07-11

**Authors:** Jevgenija Kondratjeva, Florie Julien, Céline Coutelier, Louis Humeau, Fabien Moog, Daniel Combarros, Isabelle Fourquaux, Charline Pressanti, Maxence Delverdier, Peter F. Moore, Marie Christine Cadiergues

**Affiliations:** 1 Small Animal and Equine Hospital, ENVT, Université de Toulouse, Toulouse, France; 2 Selarl Hippovet Aude, Laure Minervois, France; 3 INFINITy, Université de Toulouse, CNRS, UPS, InsermToulouse, France; 4 Centre de Microscopie Electronique Appliquée À La Biologie, Université de Toulouse, Toulouse, France; 5 Basic Sciences Department, Université de Toulouse, ENVT, Toulouse, France; 6 IHAP, Université de Toulouse, INRAE, ENVT, Toulouse, France; 7 Leukocyte Antigen Biology Laboratory, UC Davis, VM PMI, Davis, CA USA

**Keywords:** *Equus asinus*, Donkey, Skin, Cutaneous T-cell lymphoma, Clonality test

## Abstract

**Background:**

Cutaneous epitheliotropic T-cell lymphoma is a malignant tumour of the skin already reported in humans, dogs, cats, horses, and other species, but not previously in donkeys. The standard diagnosis is based on clinical, morphological and immunophenotypic data. Differentiation of malignant versus benign proliferation of lymphocytes is crucial; in ambiguous cases T-cell receptor gamma (TRG) molecular clonality should be tested. In the present paper, we report a case of mycosis fungoides diagnosed in a donkey whose diagnosis was based on clinical, histological and immunohistochemical aspects and a positive TRG clonality test.

**Case presentation:**

A twenty-five-year-old donkey gelding was referred with a mildly pruritic, generalised and severe exfoliative dermatosis. Otherwise, the animal was clinically healthy, though mildly underweight. Dermatological examination revealed severe generalised alopecic and exfoliative dermatitis, occasionally eroded, with high number of large, thin, greyish scales. All mucocutaneous junctions except the hoofs were affected. Ectoparasites and dermatophytes were ruled out. The complete blood count and blood smear evaluation revealed mild normocytic normochromic anemia. The biochemistry panel showed mild hyperproteinemia with albumin within the normal range. Protein electrophoresis showed moderate polyclonal hypergammaglobulinemia. Histological findings were characterised by interface dermatitis with massive exocytosis in the epidermis of a homogenous population of lymphoid cells showing atypia. Clusters of neoplastic cells were present within the epidermis forming Pautrier “microabscesses”. These findings are consistent with cutaneous epitheliotropic lymphoma. Immunohistochemical staining revealed uniform labelling of the neoplastic cells for CD3, and lack of expression of CD20 (a B cell lineage associated marker). Molecular clonality PCR (PARR) was performed using equine TRG primers; this revealed a clonal rearrangement in a heavy polyclonal background. Transmission electronic microscopy showed multiple lymphocytes with convoluted or cerebriform nuclei.

**Conclusions:**

This case report provides the first evidence of clinical, histopathological, immunophenotypic features, electron microscopy findings and molecular analysis of a cutaneous epitheliotropic T-cell lymphoma (mycosis fungoides) in a donkey. Our observations suggest that cutaneous T-cell lymphoma should be included in the differential diagnoses of exfoliative dermatitis, even those progressing in a chronic pattern and/or with few or no pruritus.

## Background

Cutaneous lymphomas originating from T- or B-lymphocytes are uncommon in humans, dogs, cats, horses, and other species [1–3]. They are subdivided into epitheliotropic and non-epitheliotropic types. Epitheliotropic lymphomas are typically of T-lymphocyte origin [2, 4–6] and have a variety of clinical presentations and morphological features [2, 4, 7, 8]. Amongst the three sub-forms of T-cell epitheliotropic lymphoma encountered in veterinary medicine—mycosis fungoides (MF), Sezary syndrome and pagetoid reticulosis—only the first two have been reported in horses [2, 9, 10]. The most common clinical presentation of MF in horses is exfoliative dermatosis, nodules, or ulcerations with or without pruritus, associated with infiltration of neoplastic T-lymphocytes with a specific tropism in the epidermis and adnexal structures, and oral mucosa [2, 8].

In donkeys, skin tumours are among the skin disorders reported in reviews [11, 12] or retrospective studies [13–15]. The most common neoplasia are sarcoid tumours [11–15]. To the best of our knowledge, no case of cutaneous epitheliotropic lymphoma has been described in the donkey to date. Here we report the clinical, histopathological, immunophenotypic features and positive clonality testing of a cutaneous epitheliotropic T-cell lymphoma (MF) in a donkey.

## Case presentation

A twenty-five-year-old donkey gelding was referred with a several-year history of mildly pruritic, generalised and severe exfoliative dermatosis, with exacerbation over the preceding three months. The donkey had lived in Southern France the last ten years and was kept outdoors permanently with two other donkeys in a fenced field (250 m^2^ with a 10 m^2^ cover) and occasionally with several horses from the same area; none of the in-contact animals had ever displayed a dermatological disease. The paddock was located next to a water canal where rodents were regularly seen as well as biting insects during the summer months. The owner did not report any signs of systemic illness, except mild underweight. No treatment had been given, except occasional topical applications of povidone iodine and recent topical application of deltamethrin (Butox 7.5% pour-on, MSD, Beaucouzé, France – 10 mL) following skin scrapings shown to be positive for *Ornithonyssus bacoti* mites.

**Fig. 1:**
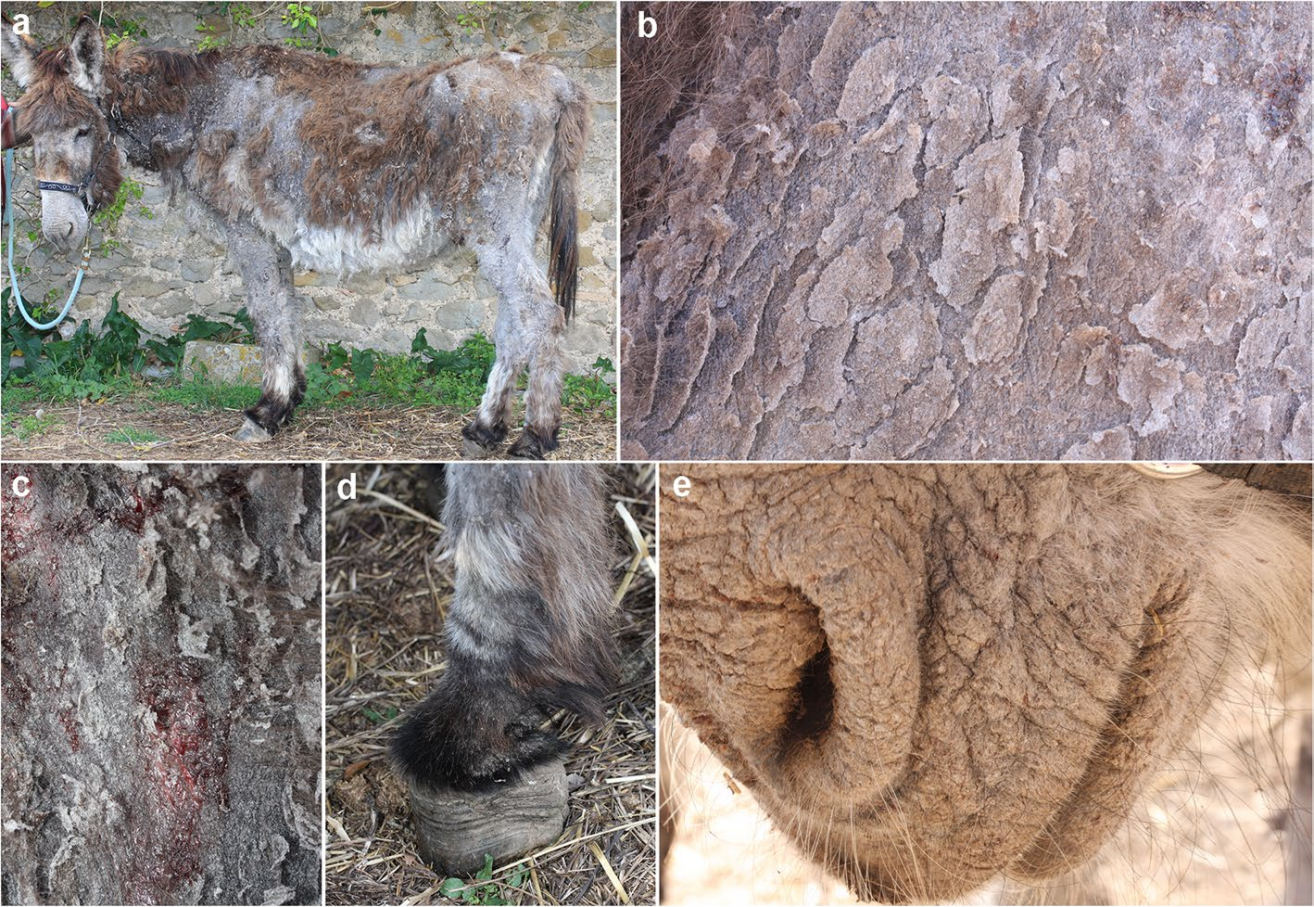
Initial physical examination. A 25-year-old donkey in poor body condition with severe generalised alopecic and exfoliative dermatitis (**a**); close up of the skin, which was generally thin, covered by large quantities of large, thin, greyish scales (**b**), occasionally eroded with sero-haemorrhagic exudate (**c**). The hoofs were not affected (**d**). The lips and muzzle were very scaly (**e**)

Physical examination on admission revealed an alert, responsive donkey with poor body condition (body score 2/5). Dermatological examination revealed severe generalised alopecic and exfoliative dermatitis (Fig. 1a). The skin was generally thin, covered by high number of large, thin, greyish scales, giving a laminated appearance upon close examination (Fig. 1b). Occasional erosions with sero-haemorrhagic exudate were present (Fig. 1c). All mucocutaneous junctions except the hoofs were affected (Fig. 1d and e).

The initial differential diagnoses included ectoparasites, dermatophytosis, pemphigus foliaceus, sarcoidosis, zinc responsive dermatosis, generalised primary seborrhoea and epitheliotropic lymphoma.

**Fig. 2:**
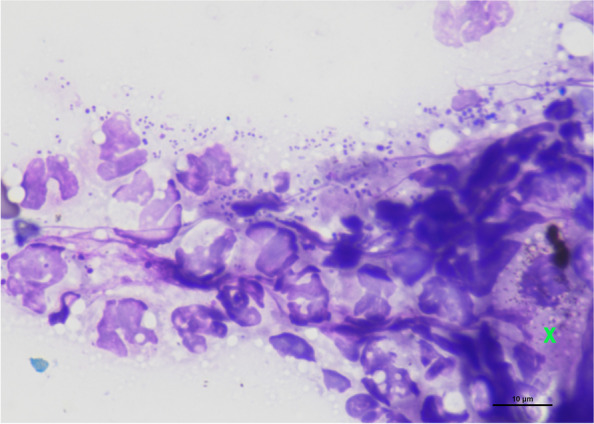
Cytological examination. A direct skin smear was taken from eroded lesions on the lateral thorax. Degenerate neutrophils, phagocytized cocci and keratinocytes (green cross) were observed. (Stained with RAL®555, RAL Diagnostics; Site Montesquieu-Martillac, France. Magnification × 1000, bar = 10 µm)

Microscopic examination of skin scrapings collected from dorsum and flanks did not reveal any remaining mites. Cytological examination of direct skin smears from eroded lesions (RAL®555, RAL Diagnostics; Site Montesquieu-Martillac, France) revealed keratinocytes, degenerate neutrophils and phagocytized cocci (Fig. 2). Bacterial culture from an exudative lesion yielded multiple colonies of *Staphylococcus hyicus* and *Streptococcus dysgalactiae*, both sensitive to all the antibiotics tested, except for *S. dysgalactiae,* which was not sensitive to gentamicin. Fungal culture of scaling material and hair shafts was negative. The complete blood count and blood smear evaluation only revealed mild normocytic normochromic anemia (4.34 10^6^ cells/μL, RI 4.4–7.1 10^6^/μL [16]). The biochemistry panel showed mild hyperproteinaemia (78.7 g/L, RI 58–76 g/L [16]) with albumin in the normal range (29.8 g/L, RI 21.5–31.6 g/L [16]). Protein electrophoresis showed moderate polyclonal hypergammaglobulinemia (39.1%, RI 8–15.8%). Coproscopic examination revealed large numbers of strongyle (1 600 eggs per gram, modified McMaster technique).

**Fig. 3:**
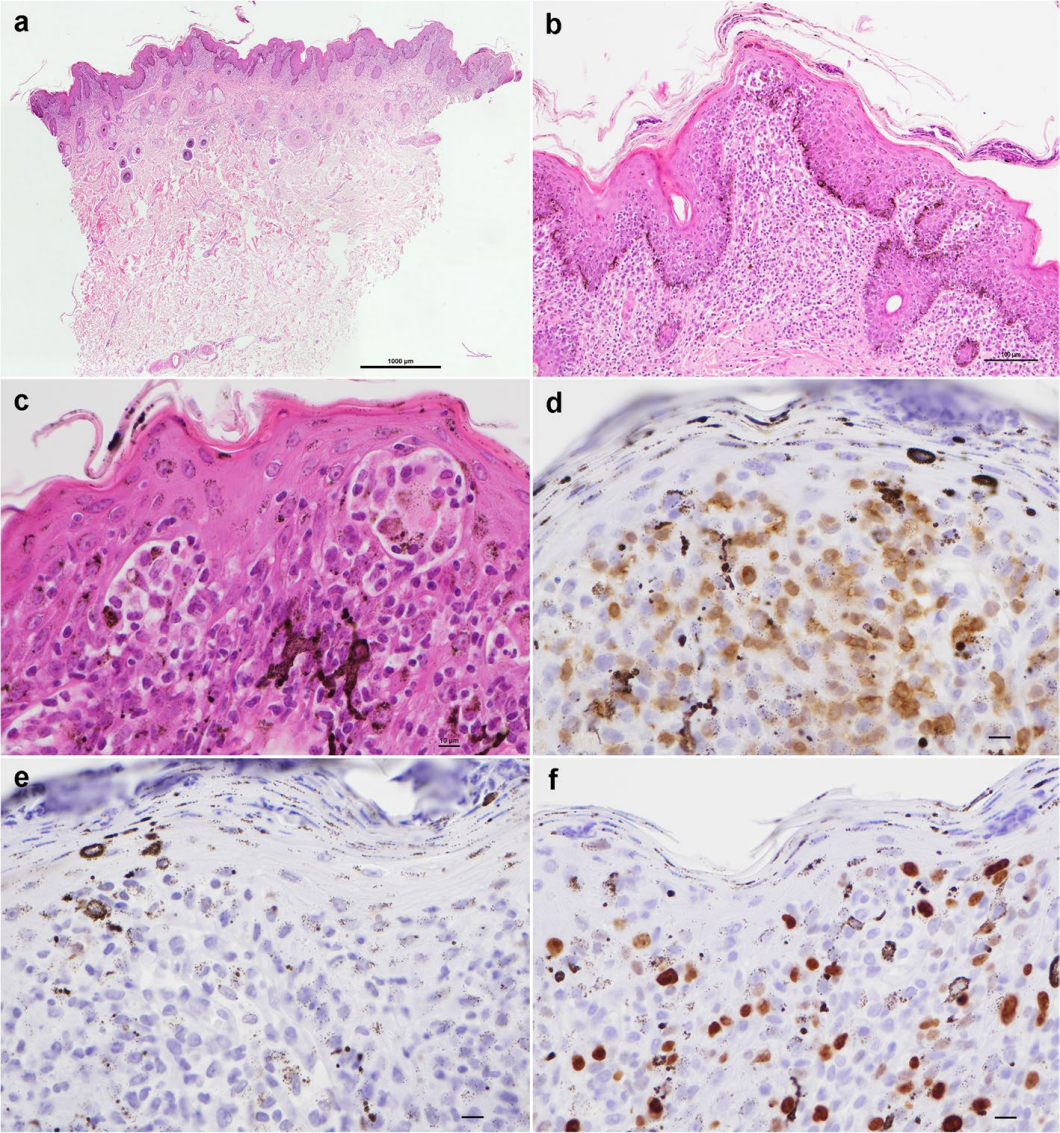
Histopathological examination of biopsies taken from the lateral thorax. Interface dermatitis (**a**) with massive exocytosis in the epidermis of a homogenous population of lymphoid cells showing atypia (**b**). Clusters of neoplastic cells were present within the epidermis forming Pautrier microabscesses (**c**); [H&E staining, magnification × 40 (**a**), × 200 (**b**) and × 400 (**c**), bars = 1000 µm (**a**), 100 µm (**b**) and 10 µm (**c**)]. Immunohistochemical staining for CD3 showed uniform labelling of the neoplastic cells for CD3 (**d**), whereas staining for CD20 was negative (**e**). The Ki-67 labelling fraction was hard to quantify in the epidermis as the background labelling of basal epithelial cells was prominent, but by comparing the labelling fraction in the superficial dermis, it was estimated at less than 20% (f). (Magnification × 400, bar = 10 µm)

Microscopic lesions in three 8-mm skin biopsy specimens from the lateral thorax were characterised by interface dermatitis (Fig. 3a) with epidermotropic lymphocytes (Fig. 3b) showing atypia in the epidermis. Clusters of neoplastic cells were present within the epidermis forming Pautrier “microabscesses” (Fig. 3c).

Immunohistochemical staining for CD3 (réf M7254, clone F7.2.38, dilution 1/50, Agilent Technologies, Les Ulis, France), CD20 (réf PA5-16,701, polyclonal, dilution 1/600, Invitrogen, Thermofisher Scientific, Waltham, Massachusetts, USA) and Ki-67 (réf M7240, clone mib1, dilution 1/50, Agilent Technologies, Les Ulis, France) revealed uniform labelling of the neoplastic cells for CD3 (Fig. 3d), and lack of labelling for CD20 (Fig. 3e). The Ki-67 labelling fraction was hard to quantify in the epidermis as the background labelling of the basal epithelial cells was prominent, but the labelling fraction in the superficial dermis was estimated at less than 20% (Fig. 3f).

**Fig. 4:**
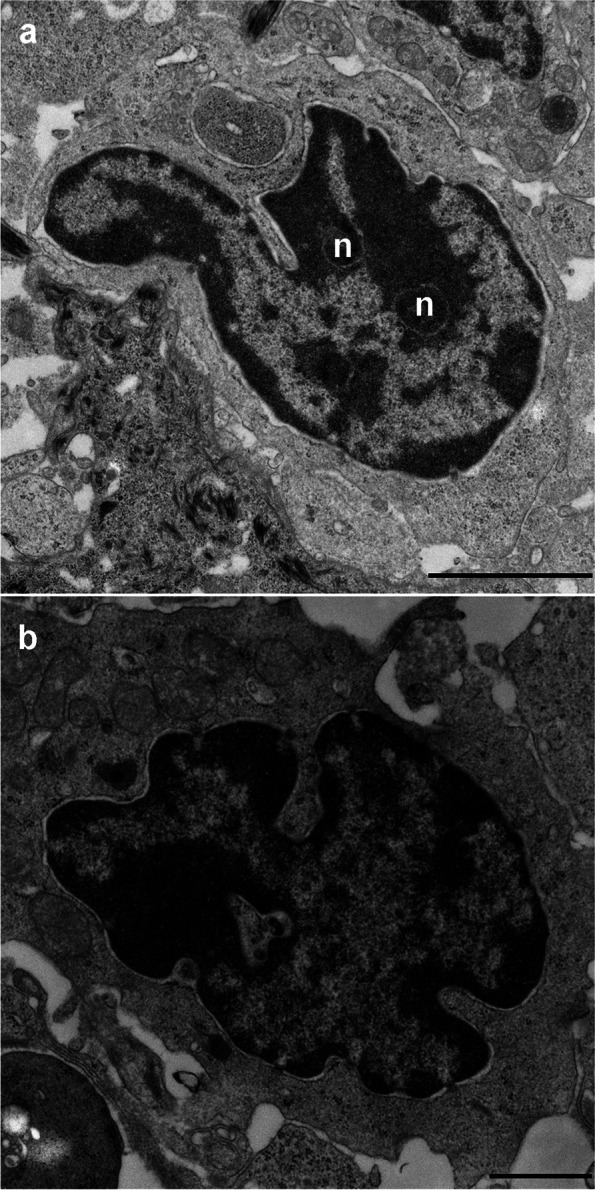
Electron microscopy of a skin biopsy taken from the lateral thorax showing multiple lymphocytes with convoluted (**a**) or cerebriform (**b**) nuclei. Prominent nucleoli were observed in the nucleus (n). Scale bars = 1 µm

Electron microscopic examination of lesional skin revealed multiple lymphocytes with convoluted (Fig. 4a) or cerebriform (Fig. 4b) nuclei. Prominent nucleoli were observed (Fig. 4a).

**Fig. 5:**
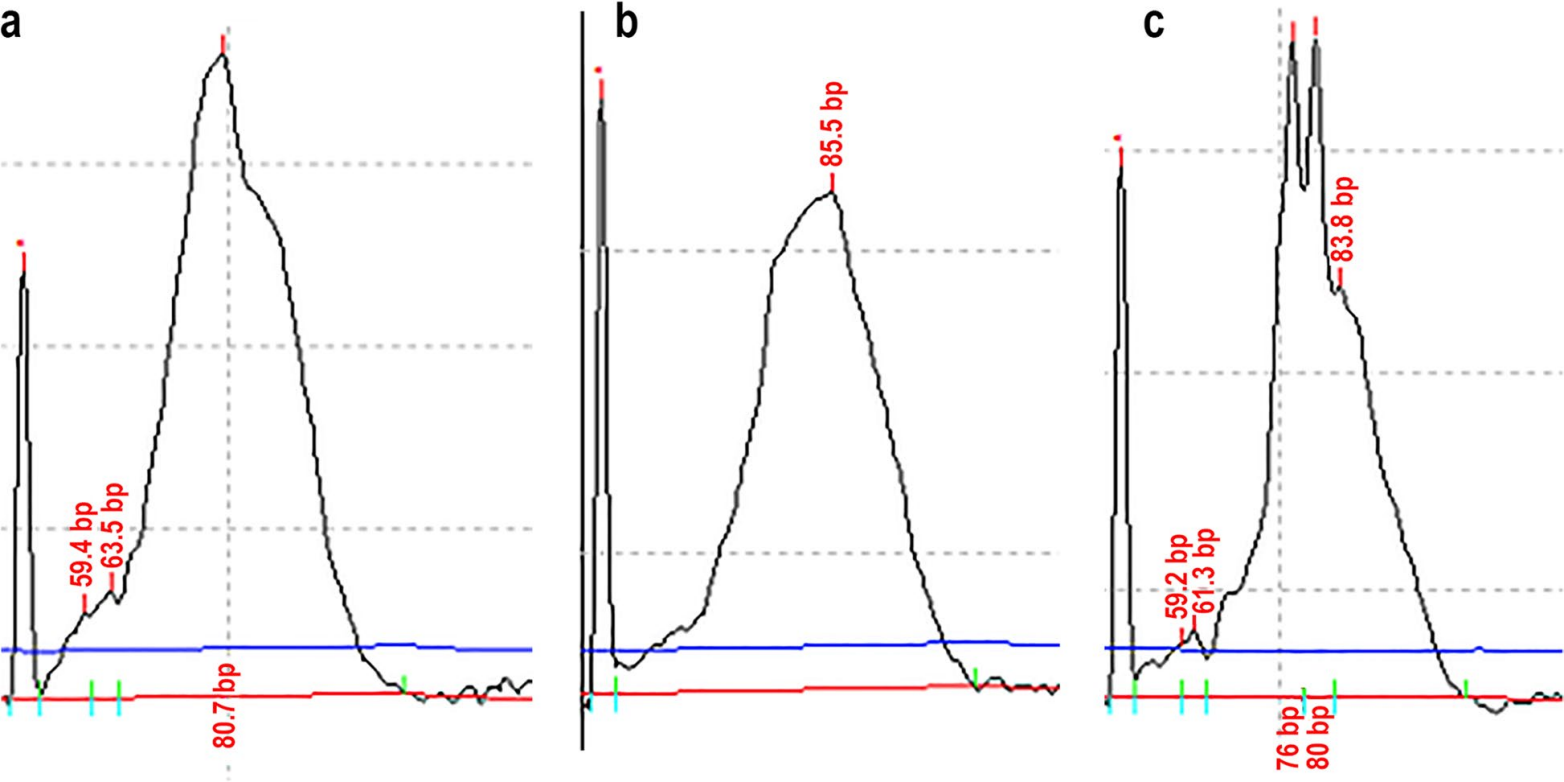
Capillary electrophoresis traces of PCR for antigen receptor rearrangement (PARR). T-cell receptor gamma genes (TRG) rearrangement analysis of blood from a normal 8-month old donkey (**a**), blood (**b**) and skin (**c**) of the donkey with mycosis fungoides. a and b: TRG polyclonal; c:. TRG clonal in a polyclonal background—clonal peaks are at 76 bp and 80 bp. The left most peak in all trace files (**a**, **b** and **c**) is the 50 bp calibration marker

Molecular clonality PCR (PCR for antigen receptor rearrangement, PARR) was performed using equine primers (UC Davis Leukocyte Antigen Biology Laboratory proprietary) as no primer was available for donkeys. The DNA extracted from the blood from a normal 8-month old donkey (Fig. 5a) and from the buffy coat smears of the donkey’s peripheral blood (Fig. 5b), which served as polyclonal controls for TRG, produced a symmetrical polyclonal amplicon. In the skin of the donkey with mycosis fungoides, clonal rearrangements of TRG were observed in a heavy polyclonal background (Fig. 5c). Clonal peaks were at 76 bp and 80 bp.

Taken together, the clinical appearance, the histological findings, the immunophenotypical features and the positive T-cell clonality test, confirmed our hypothesis of a cutaneous epitheliotropic T-cell lymphoma (MF).

As the donkey was considered comfortable enough, the owners did not wish to attempt any specific treatment. Only skin care, alternating kerato-modulatory (Douxo®S3 Seb, CEVA, Libourne, France) and antiseptic (Douxo®S3 Pyo, CEVA, Libourne, France) once weekly shampooing was recommended after clipping the non-alopecic areas. A fortnightly application of delamethrin (Deltanil®10 mg/mL, Virbac, Carros, France—10 mL) was prescribed to *(i)* limit fly populations and the possible development of myiasis on the eroded zones and *(ii)* prevent *O. bacoti* reinfestations. Fenbendazole (Panacur pâte, MSD, Beaucouzé, France) was given orally to the two other donkeys at the manufacturer’s recommendation dosage.

One year after diagnosis, at the time of writing, the donkey is still alive, and the lesions appear to be stable.

## Discussion and conclusion

Progression of the cutaneous disease over several years had been reported by the owner. Although the animal had been examined several times by veterinarians, no further investigation had been undertaken except recent skin scrapings which had revealed *O. bacoti* infestation. Hence, the speed of progression of the condition is difficult to evaluate. In humans and dogs, MF typically progresses through several distinct or overlapping clinical stages consisting of patch and plaque formation, followed by subsequent progression to the tumour stage with potential for metastasis to regional lymph nodes and visceral organs [3, 4]. The progression of MF to fatal disease in humans may take years or even decades [17]. In a retrospective study of 30 cases in dogs, the mean time between the first onset of lesions and the final diagnosis of cutaneous epitheliotropic T cell lymphoma was 5.5 months [4]. Slowly progressive disease has also been reported in cats [5]. Previous persistent antigenic stimulation or chronic inflammation might predispose to development of MF in both humans and animals, diagnoses such as atopic dermatitis, psoriasis or infections have been implicated, but this link remains the subject of debate [1, 4, 18, 19].

The accurate distinction between reactive and neoplastic lymphoid proliferations and the differentiation of T- and B-lymphocytes can be challenging, all the more so since in horses, the most frequent cutaneous malignant lymphomas are T-cell-rich B-cell lymphomas [9, 10, 20]. Molecular clonality assays, assessing clonality of T- or B-cells through PARR can effectively complete clinical, morphologic, and immunophenotypic assessments [21, 22]. Clonality assays are species-specific and in veterinary medicine, are currently only available for dogs, cats, and horses [21–23]. In the present case, the test was performed using primer sets for equine PARR on formalin-fixed paraffin-embedded tissue. To the authors’ knowledge, this is the first documented use of PARR in donkeys.

At the time of diagnosis, mild normocytic normochromic anemia was observed. This abnormality is not reported in MF without lymph node involvement in humans, or in animals [5, 10, 17, 24–26]. The recent infestation by tropical rat mites possibly explains the mild anemia as these mites are hematophagous and in case of a heavy infestation, can cause anemia [27].

Skin-directed and systemic treatments of MF are clearly described in human medicine, depending on the stage, the areas affected and systemic involvement. Treatments include topical steroids, phototherapy, electron beam therapy, radiation, retinoids, monoclonal antibodies, single and multiagent chemotherapy [28, 29]. In horses, data concerning T-cell lymphoma are limited to one horse with a nodular form which was treated with intralesional injections of betamethasone associated with megestrol acetate tablets in addition to surgical removal of ulcerated lesions [30].

In conclusion, this case report provides the first evidence of clinical, histopathological, immunophenotypical features, electron microscopy findings and molecular analysis of a cutaneous epitheliotropic T-cell lymphoma (mycosis fungoides) in a donkey. Cutaneous T-cell lymphoma should be included in the differential diagnoses of exfoliative dermatitis, even with a prolonged time course and little or no pruritus.

## Data Availability

The data generated, and/or used during the work-up of this case cannot be made publicly available in the interests of retaining patient confidentiality but are available from the corresponding author on reasonable request.

## Abbreviations

CD: Cluster of differentiation
TRG: T-cell receptor gamma genes
RI: Reference interval
PARR: PCR for antigen receptor rearrangement
DNA: Deoxyribonucleic acid
MF: Mycosis fungoides
bp: Base pairs
